# The route to microstructures with optical quality glass surfaces by fs laser ablation

**DOI:** 10.1038/s41598-025-11563-0

**Published:** 2025-07-14

**Authors:** Dominika Schrödter, Patryk Mietliński, Bartosz Gapiński, Andreas Dietzel

**Affiliations:** 1 Institute of Microtechnology (IMT), Faculty of Mechanical Engineering, Technical University of Braunschweig, Alte Salzdahlumer Straße 203, 38124 Braunschweig, Germany; 2 Institute of Mechanical Technology, Faculty of Mechanical Engineering, Poznan University of Technology, ul. Piotrowo 3, Poznań, 61-138 Poland

**Keywords:** Femtosecond laser ablation, Borosilicate glass, Roughness analysis, Surface morphology, Materials for optics, Laser material processing

## Abstract

Glass substrates are highly valued for their transparency, stability and biocompatibility, making them essential in optics, photonics, chemistry and biomedical devices. Femtosecond lasers enable precise glass ablation due to the capability to deliver pulses with high energy density leading to nonlinear absorption. This study examined how parameters like pulse energy, scan line density and pulse repetition rate affect the surface roughness of 3D borosilicate glass structures. Analysis by means of AFM, µCT and SEM revealed parameter settings to minimize roughness and prevent substrate damage. Post-treatments like HF etching and annealing further enhanced surface quality, resulting in smooth, transparent (T = 85.35%) microstructures especially for novel optical and optofluidic applications.

**Supplementary Information:**

The online version contains supplementary material available at 10.1038/s41598-025-11563-0.

## Introduction

Femtosecond (fs) laser microfabrication has recently emerged as a powerful technique for microfabricating and microstructuring across both opaque and transparent materials such as metals, biomaterials, polymers and brittle materials^1–5^. The unique characteristics of ultra-short laser pulses in the femtosecond range (10^–15^ s) offer advantages such as minimal heat-affected zones, high precision and the ability to achieve sub-micron feature sizes. Femtosecond laser micromachining, as a direct maskless processing technology, offers unparalleled capabilities for 3D fabrication of micro-optical and microfluidic devices compared to other established micromachining techniques such as photolithography and etching or soft lithography^6,7^. The use of femtosecond laser processing has been investigated in recent years for the fabrication of transparent microfluidic structures and devices^8–10^. Local nonlinear absorption in very confined volumes of optically transparent materials facilitates the structuring with high resolution with more 3D freedom compared to conventional microfabrication techniques^11^. Combined with laser beam scanning or sample translation, it enables to micromachine geometrically complex structures of transparent materials at micro and nano-level of accuracy in three dimensions^12,13^. Thus, fs laser ablation has a great potential in the fabrication of miniaturized glass devices, with applications in various fields including optics, as well as fluidics for biological and chemical processes^14–16^.

Microchannels fabricated on glass play an increasingly important role in the miniaturization of microfluidic devices due to the precision and repeatability essential for high-quality microfluidic device production and possibility to reuse systems repeatedly for iterative studies. Moreover, high transparency, chemical stability and biocompatibility of glass substrates are superior relative to other materials. However, glass as a substrate material, also poses technical challenges for laser processing due to its high hardness, great brittleness and low optical absorption^17^. Glass-based microfabrication demands meticulous parameter selection to mitigate issues such as surface roughness, microcracks and the formation of undesirable recast layers^18^.

Establishment of relationship between fs laser microfabrication parameters and surface quality is crucial to manufacture high quality glass microfluidic chips. Erfle et al. presented a gas/liquid Taylor flow micromixer made of glass for stabilized production of lipid nanoparticles of tunable size in Taylor flow devices^19^. It was demonstrated that reduced surface roughness of the channel walls prevented the oil droplets from being deposited in the existed cavities on the channel walls and reducing fouling, thus allowing the long-term operation of the microdevices. Hanada et al. showed biochip as a nanoaquarium fabricated in Foturan glass by femtosecond laser, which was produced to observe microorganisms such as E. gracilis^20^. It was reported that surfaces of the inner wall typically had a roughness, which causes scattering of light and distortion of the wavefront. With adjusted fabrication procedure by multiple scanning of the laser beam with lateral shifts and post additional annealing, it resulted in flat and smooth surfaces, that enabled to capture images of the E. gracilis swimming in the microchannel by microscopic observation. Microfluidic components often require a low roughness and high accuracy for targeted fluidic stimulation, advanced microscopy and long term cultivation of living tissue. Research has shown that both chemical and thermal treatments can significantly impact the properties of glass after laser ablation^21–23^. The combination of various post treatments can lead to favorable surface conditions, reducing micro-defects and particle deposition on the surface, thus enhancing the surface quality after laser ablation.

In our previous work, it was reported that using femtosecond laser ablation technique a monolithic microfluidic glass chip that can be used in combination with a commercial light-sheet microscope has been microfabricated^24,25^. This has already shown the importance of a suitable femtosecond laser ablation process to create windows for two sheets of light from opposite directions that overlap perfectly, so that the focal plane of the imaging lens has been precisely defined, so that the imaging quality enables a resolution capability for recording single cell responses from different parts of the Danio rerio brain. However, a systematic study of parameters for laser ablation, resulting in most perfect surface qualities in terms of roughness and transparency was still missing.

The primary objective of this study was therefore to systematically investigate how variations in femtosecond laser parameters such as pulse energy, number of scanning lines and pulse repetition rate affect the surface roughness and morphology of the 3D structured glass surfaces. The surface morphology of glass ablated within parameter ranges was studied and the roughness of the surface was measured by AFM, µCT scans and SEM to provide the complex surface characteristic. The surface characteristics and surface roughness will be discussed in details, including chemical and thermal post processing and its impact on the surface quality after iterative laser ablation. This knowledge will enable precise control over surface quality, leading to improved performance of glass-based devices across various fields.

## Materials and methods

### Microstructuring

4-inch size Borofloat^®^ 33 borosilicate 1.1 mm thick glass wafers (Schott, Mainz, Germany) were used as a substrate for ablating parameter slots. For microstructuring, a femtosecond laser microstructuring system (microSTRUCT c, 3D Micromac AG, Chemnitz, Germany) equipped with a YB: KGW laser source (Pharos, Light Conversion, Vilnius Lithuania) was used. It was operated at the fundamental wavelength of 1030 nm, emitting pulses of 227 fs and 212 fs at a pulse repetition frequency of 100–600 kHz, respectively. Using a F-theta lens with a focal length of 100 mm, the beam was scanned over the substrate surface by a galvanometer scanner (Scanlab RTC5, Puchheim, Germany). The parameter slot design (by AutoCAD 2015 resulting in DXF-file) was first imported into laser software (MicroMMI from 3D–Micromac) and depending on the specific parameter study, resulting in a single layer of 50 μm after laser processing. In order to study the influence of the chemical and thermal post treatment on surface roughness after iterative laser ablation, the complex design was sliced into 14 layers of 50 μm and microfabricated, resulting in channels with a depth of 700 μm.

### Femtosecond laser ablation parameters

Different laser power levels expressed by the high voltage of the pulse picker (HVpp) varied from 1000 V to 2200 V (Supplementary Data - Table 1) were studied. The values of the average laser power $\:{P}_{avg}$ at different HVpp were measured during the laser ablation process and the corresponding pulse energy *E* was calculated for pulse repetition rate $\:{f}_{rep}=100\:kHz$ and $\:{f}_{rep}=600\:kHz\:$as

1 $$\:E=\frac{{P}_{avg}}{{f}_{rep}}$$

Using pulse energy $\:E$ and the area $\:A$ over which the energy was distributed (the laser spot diameter was defined^26^ and equal $\:D=34\:\mu\:m$ for $\:\lambda\:=1030\:nm$), the laser fluence was calculated (Table 1) as

2 $$\:F=\frac{E}{A}\:[\text{J}/{cm}^{2}]$$

**Table 1:** Parameters used for the femtosecond laser ablation of studied parameters slots within the base glass substrate.

High voltage of the pulse picker (HVpp) [V]	Fluence at 100 kHz (J/cm^2)^	Fluence at 600 kHz (J/cm^2)^
1000	6.19	1.03
1100	7.27	1.21
1200	8.36	1.39
1300	9.44	1.57
1400	10.45	1.74
1500	11.40	1.90
1600	12.28	2.05
1700	13.04	2.17
1800	13.70	2.28
1900	14.21	2.37
2000	14.68	2.45
2100	15.03	2.50
2200	15.20	2.53

**Fig. 1:**
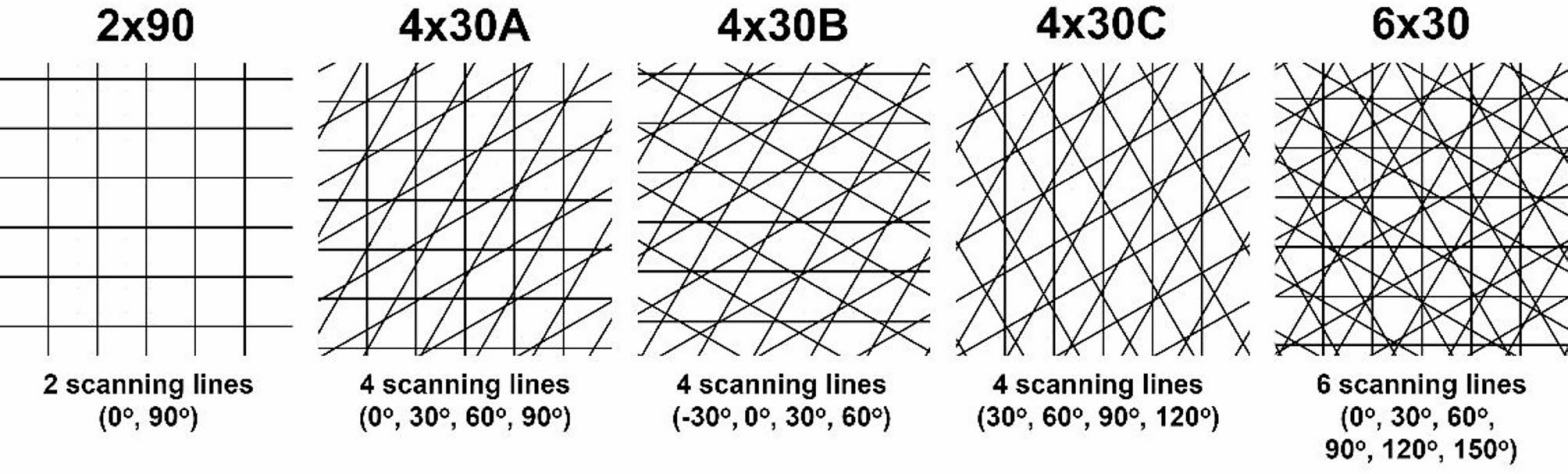
Filling strategies with different number and direction of scanning lines, starting with two (2 × 90), four (4 × 30 A-C) and six (6 × 30) scanning lines. The parameter slot design was 1.5 × 1.5mm^2^ square and the distance between scanning lines was 4 μm.

Five filling patterns (Fig. 1), that differed in the number and the direction of scanning lines, were used to create 3D structures in a layer-by-layer fashion of ablation.

### Scanning electron microscope (SEM) imaging

Morphological observation of parameter slots coated with a thin gold layer were performed by SEM (Phenom XL, LOT-Quantum Design GmbH, Darmstadt, Germany) at 15 kV accelerating voltage using the backscattered electron detector.

### Atomic force microscope (AFM)

Using optical 3D surface profilometers can lead to artefacts in the evaluation of surface quality of transparent materials. Therefore, AFM was considered as the better alternative^27^. For surface roughness evaluation, studied parameter slots were single layer groove with 50 μm in depth and the AFM (Nanosurf, NaniteAFM, Switzerland) could be used to characterize the surface roughness regarding to increasing laser energy and different filling strategies. The AFM measurements were conducted in dynamic force mode using a monolithic silicon cantilever Tap190AI-G. Considering the distance between scanning lines (4 μm apart from each other), AFM scan area was set to 50 μm x 50 μm, so that area to measure include at least 10 scanning lines in x and y direction for each filling strategy. The AFM analysis was always performed in the centre of studied parameter slots. The surface roughness was quantified using the MountainsLab^®^ software, providing detailed insights into the surface structures. The root mean square deviation of the surface roughness height from the reference plane (Sq) and the arithmetic mean deviation of the surface roughness height from the reference plane (Sa) parameters were used to specify machined surfaces. These parameters are used generally to evaluate surface roughness and represent an overall measure of the texture comprising the surface.

### Optical profile measurements

The analysis for each parameter combination including different laser power and filling strategy was performed with a confocal laser scanning microscope (LSM) (KEYENCE Deutschland GmbH, VK-X260, Neu-Isenburg, Germany). The side wall angle of the parameter slots (500 μm in depth) to the glass plane (taper angle) were evaluated using 10x magnification. Both the differential interference contrast (DIC) and optical mode were used for defects evaluation.

### X-ray micro-computed tomography (µCT)

Computed tomography (CT) is a non-invasive diagnostic technique using X-ray radiation to examine internal structures and complex geometries^28–30^. The v|tome|x s240 CT scanner (Waygate Technologies) was used with settings of 100 kV, 150 µA, 300 ms exposure, 1.8 μm voxel size, and 1400 images. Tomographic analyses were conducted using Volume Graphics MAX 2.2 software. This method enabled detailed surface analysis of small glass samples in challenging areas, such as 700 μm deep channels. Due to the material’s specific properties and the sample’s geometric shape, surface quality after iterative laser ablation could not be determined using other measurement techniques. The sample was placed on a rotating platform, capturing X-ray images at multiple angles to generate a 3D model analysis of geometric features and surface analysis.

### Surface analysis

The surface measurement after chemical and thermal post-treatment was conducted using micro-CT, the only method enabling full surface topography visualization. Optical and scanning methods were unsuitable due to the sample’s deep topography (700 μm channels) and material transparency. X-ray images were reconstructed in Phoenix Datos|x2 and analyzed in Volume Graphics MAX 2.2, generating a 3D STL model for further analysis in GOM Inspect V8 and MountainsMap 10 (roughness parameters were calculated according to ISO 25178, ISO 16610, and ISO 13565). Roughness parameters (Sa, Sq, Sz) provided a general but unreliable representation of surface texture. After laser ablation, the Sa and Sq parameter were used for analysis to indicate the general effect on surface quality. In contrast, after chemical and thermal treatment, the Sa and Sq parameter did not highlight significant changes in topography. For a more detailed analysis, it was necessary to check the values of other parameters that could indicate surface transformation. According to the analysis and obtaining images from the SEM (Fig. 5B), an unambiguous transformation in the context of surface isotropy was noted. The possible characteristics of these transformations are confirmed by the publication of Pawlata^31^. Standard metrics failed to distinguish geometric changes, making them insufficient for detailed analysis. Other authors such as Blaineau et al.^32^ have used other parameters capable of illustrating these changes. Instead, the Texture Aspect Ratio (Str) was identified as the most accurate parameter for assessing surface isotropy changes, with values ranging from 0.00 (high directionality) to 1.00 (isotropic texture). The Str parameter effectively detected modifications due to technological processes, offering a precise evaluation of surface transformations.

### Optical transparency analysis

**Fig. 2:**
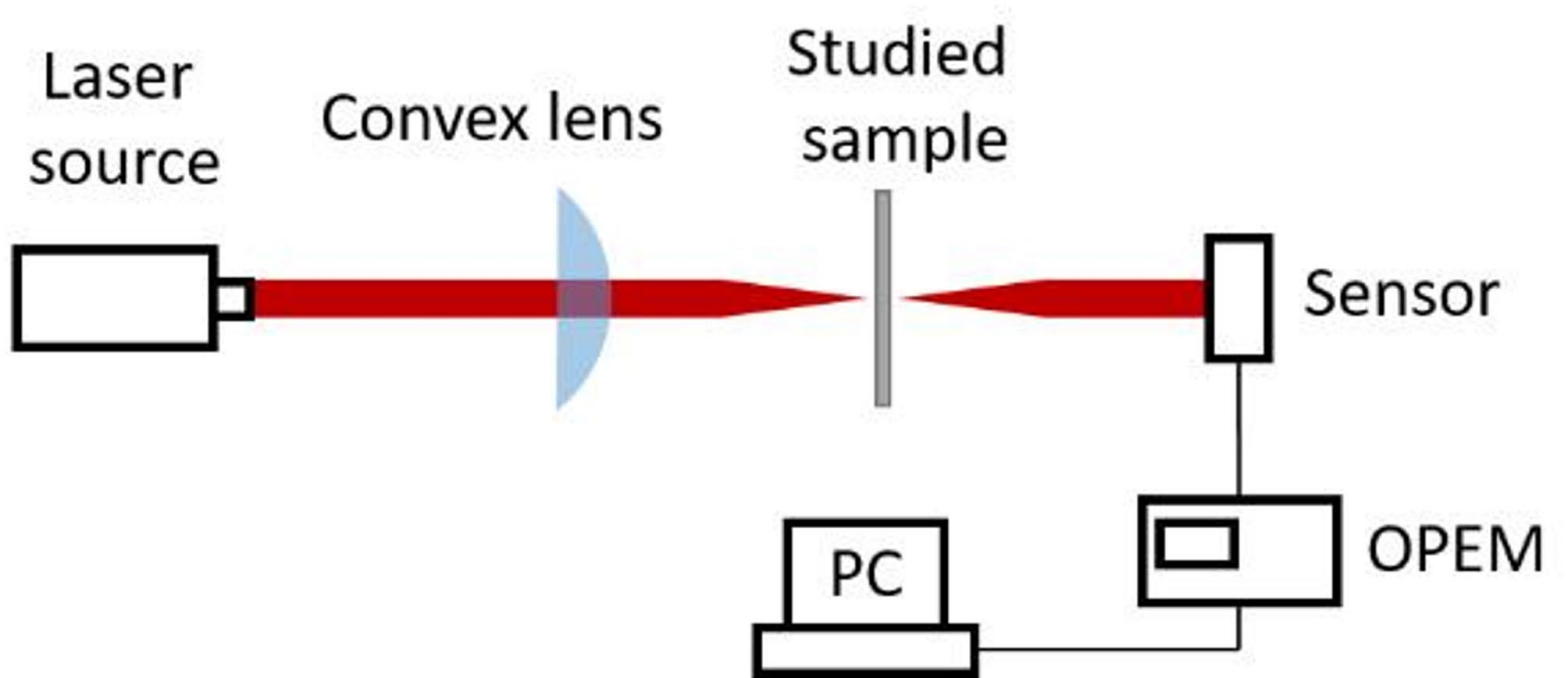
Schematic of light transmittance measurement setup.

Using laser light at a wavelength of 1550 nm, intensities of transmitted light were measured to evaluate the transmittance of glass surfaces after femtosecond laser ablation. The laser beam was focused to a small spot using a convex lens with a focal length of 19 cm and the light transmitted on the optical axis was measured by the optical power and energy meter (OPEM) at a distance of 4 cm behind the focal point (Fig. 2). After measuring the power of unscattered light *P*_*i*_ = 4.979mW, the glass samples were placed at the focal position and optical power *P*_*t*_ influenced by the glass samples with varied surface conditions was measured. The measurements were carried out in a dark box to avoid the influences of ambient light.

## Results

### Effect of laser energy and filling strategy on taper angle

**Fig. 3:**
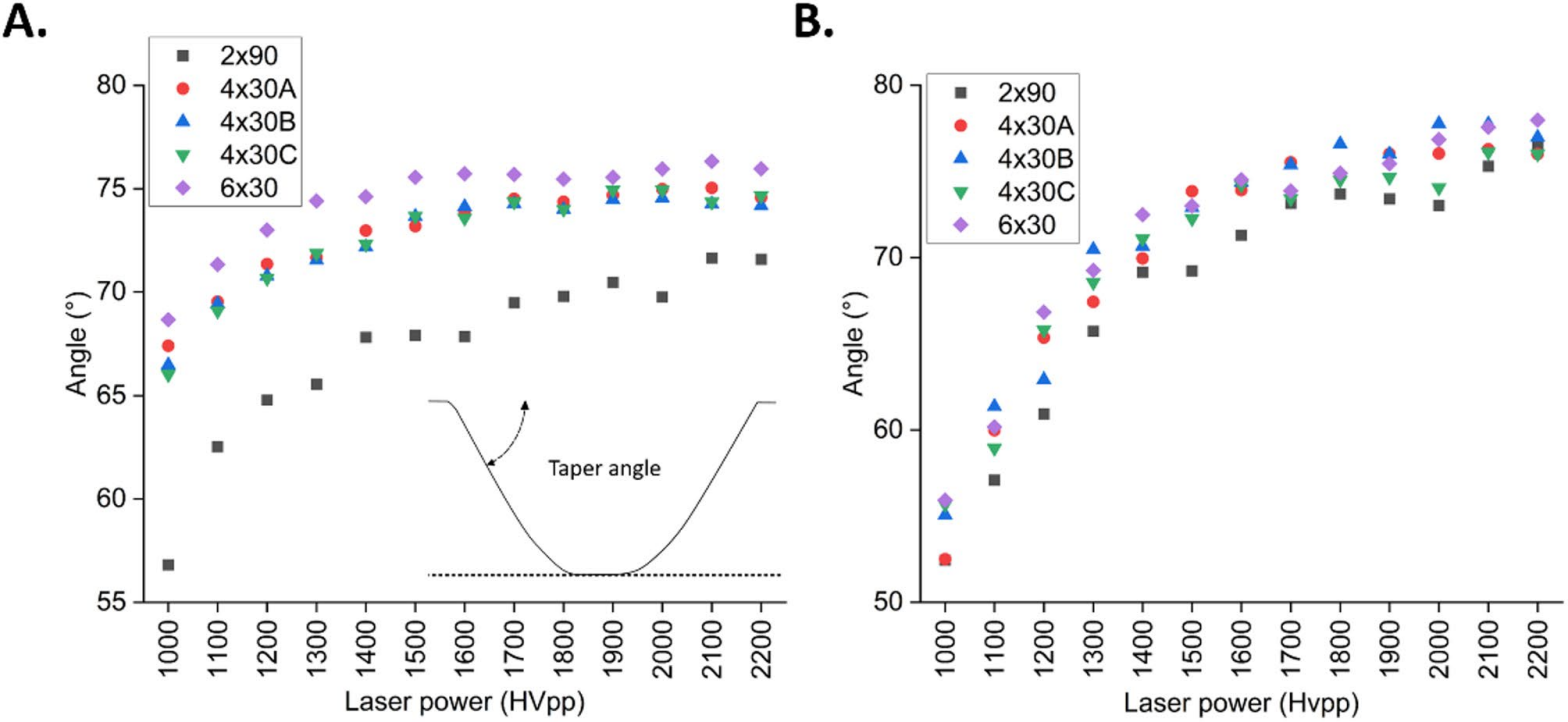
Effect of laser energy and filling pattern on wall taper angle at pulse repetition rates of 100 kHz (**A**) and 600 kHz (**B**). The inserted schematic illustrates the taper angle definition.

The dependencies of wall taper angle on different filling strategies and on pulse repetition rates are shown in Fig. 3. The taper angle was measured using a laser scanning microscope (LSM) as the angle between the wafer surface and the sidewall of the ablated slot. It varied between 57° and 77° for 100 kHz and between 52° and 78° for 600 kHz. For all filling strategies the taper angles increased with rising laser power. With the 6 × 30 filling strategy the largest angles could be reached. The ablation depth achieved in each case is probably the main influencing factor which also explains the differences observed for different repetition frequencies. Deeper ablation tends to show bigger taper angle due to Gaussian intensity profile of the laser beam, where the intensity is the highest at the center and decreases toward the edges. As a result, the material removal is more pronounced at the center of the beam, leading to tapered sidewalls.

### Effect of laser energy and filling strategy on surface roughness

**Fig. 4:**
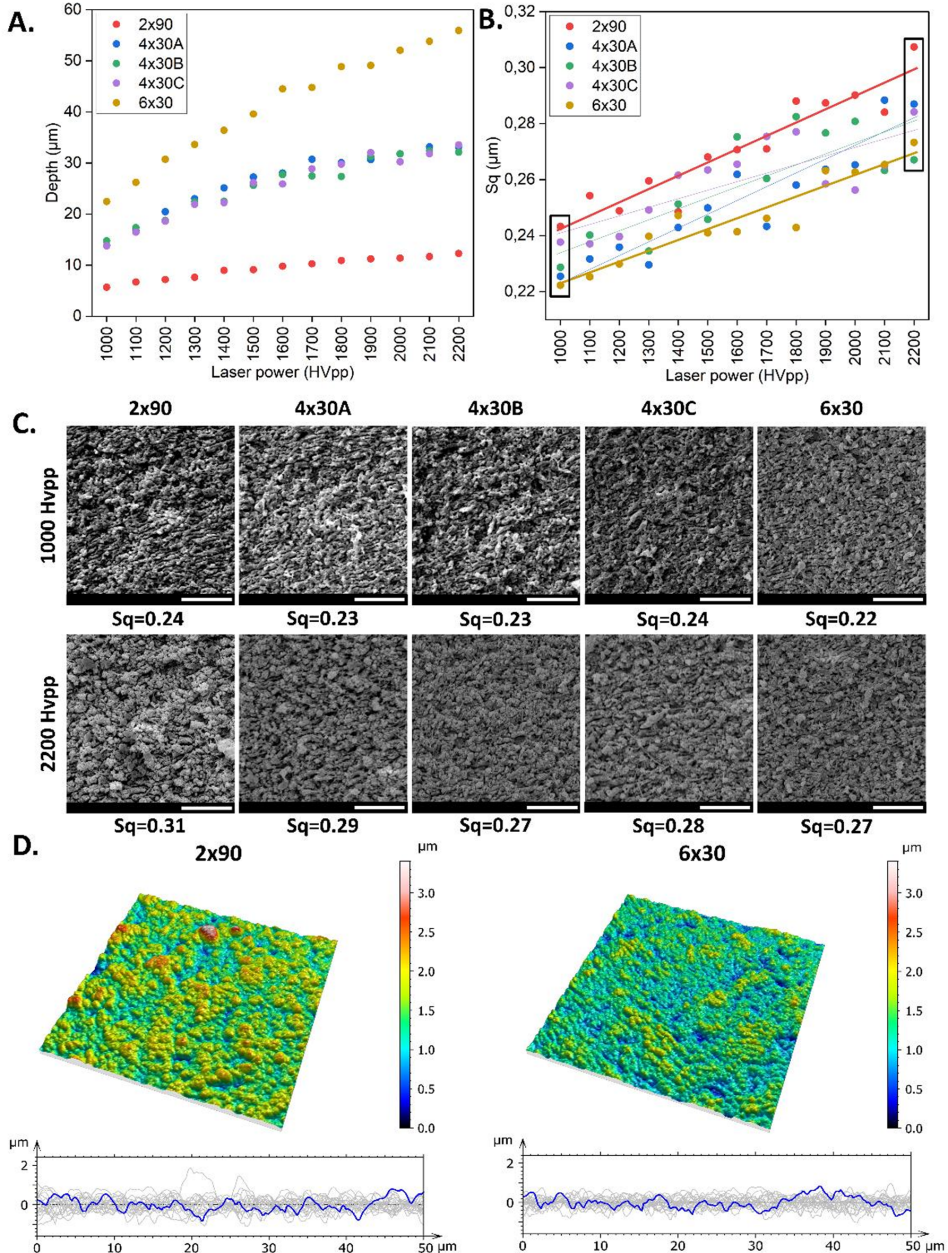
Effect of laser energy and filling strategy on removal depth (**A**) and on surface roughness (**B**) using a pulse repetition of 100 kHz. Surface roughness expressed by average roughness Sq for five studied filling strategies (2 × 90, 4 × 30 A-C, 6 × 30) at different laser power settings. Comparison of surface roughness for 2 × 90 and 6 × 30 at different laser power settings were highlighted with bold red and yellow lines. (**C**) Exemplary SEM (for data marked by two vertical rectangles at HVpp = 1000 V and HVpp = 2200 V in Fig. 3B) illustrate surface improvements after increasing number of scanning lines. The scale bar is 8 μm. (**D**) Comparison of topography maps and roughness profiles of 2 × 90 and 6 × 30 filling strategy at HVpp = 2200 V laser power. Topography maps obtained with filling strategies of two and six scanning lines illustrate the influence on the surface quality. A series of profiles is shown as grey lines, while blue profiles represent averaged profiles.

Laser power is a critical parameter that determines how much material is removed by ablation and consequently also affects the surface roughness. Higher laser power can remove more material per pass, leading to more significant roughness. The roughness trend indicated that the higher laser power has led to increased surface roughness, due to deeper ablation craters forming on the surface. For all studied filling strategies, the roughness was evaluated using AFM and increased as the laser power increased (Fig. 4B; Table 2). Low laser power resulted in surfaces with low roughness due to the minimal and precise material removal. It enabled finer control over the ablation process, leading to a more uniform and smoother surface topography. Figure 4C shows significant changes in roughness of the surface, which was also indicated by the Sq parameter values. While operating at high laser power, coarser surface features were created due to the high energy impact, resulting in increased surface roughness. It led to uneven material removal, causing surface irregularities and higher roughness. Low laser power ranges allow for gentle, controlled material removal and consequently provided uniform features with smoother surfaces, whereas high laser power enables rapid and aggressive ablation with pronounced features and rougher surface, and additionally could cause potential melt.

**Table 2:** The root mean square (Sq) values for studied filling strategies and repetitions rate. Please note that only for 2 × 90 filling strategy, surface roughness was also measured for repetition rate of 600 khz. In all other filling strategies, the pattern obtained at 600 khz.was too deep below the substrate surface to come in contact with AFM tip.

Laser power	The root mean square (Sq) (µm)					
	2 × 90		4 × 30 A	4 × 30B	4 × 30 C	6 × 30
	600 kHz	100 kHz	100 kHz	100 kHz	100 kHz	100 kHz
1000	0.1965	0.2433	0.2255	0.2287	0.2377	0.2223
1100	0.2093	0.2543	0.2317	0.2402	0.2371	0.2253
1200	0.2031	0.2489	0.2359	0.2397	0.2396	0.2299
1300	0.2330	0.2596	0.2296	0.2345	0.2492	0.2398
1400	0.2228	0.2485	0.2429	0.2513	0.2616	0.2472
1500	0.2368	0.2681	0.2499	0.2458	0.2635	0.2410
1600	0.2583	0.2707	0.2619	0.2753	0.2655	0.2414
1700	0.2573	0.2710	0.2433	0.2604	0.2754	0.2462
1800	0.2408	0.2881	0.2581	0.2825	0.2771	0.2429
1900	0.258	0.2874	0.2637	0.2767	0.2585	0.2632
2000	0.2794	0.2902	0.2653	0.2808	0.2563	0.2627
2100	0.3082	0.2841	0.2884	0.2633	0.2650	0.2654
2200	0.3183	0.3074	0.2870	0.2671	0.2842	0.2733

While keeping the number of scanning passes constant, the orientation, which was varied between filling strategies 4 × 30 A, B and C, did neither have an effect on ablation depth nor on surface roughness (Fig. 4A and B). However, the number of scanning passes of a laser beam directly impacts surface roughness and ablation depth. With more passes at different orientations, the surface shows less of the texture patterns of the fill lines, which reduces the roughness. Upon comparing the roughness, 6 × 30 filling strategy represented lower roughness and exhibits a reduction in Sq from Sq = 0.24 μm to Sq = 0.22 μm for HVpp = 1000 V and from Sq = 0.31 μm to Sq = 0.27 μm for HVpp = 2000 V. Comparing topography maps (Fig. 4D) of filling strategies with 2 or 6 scanning lines at the same laser power (HVpp = 2200 V), less uniform texture and roughness across the surface was observed for filling 2 × 90. Just with two scanning lines, insufficient overlap could result in higher roughness. The surface showed irregularities and unevenness with significant peaks and valleys. Filling 6 × 30 with higher number of scanning lines could lead to more uniform texture and roughness across the surface, as irregularities from the initial pass were mitigated with the overlapping lines. Additional passes could smooth out the surface by removing or reducing these irregularities, reducing surface roughness. Surface features have been evened out by gradually removing asperities (high points) and filling in valleys (low points).

Analysing height variations along a surface, the roughness profile of 2 × 90 filling indicated a coarse texture, probably resulting from aggressive machining parameters. A high peak-to-valley height (Rz) of 1.01 μm suggests significant surface irregularities. Uneven peaks and troughs distribution could indicate non-uniform ablation due to high energy effects concentrated in fewer number of scanning lines. Upon comparing the roughness profiles, 6 × 30 filling strategy exhibits a reduction in Rz to 0.79 μm, highlighting the modification of surface features due to increased number of scanning lines. Compared to the filling strategy 2 × 90, surface proceed by 6 × 30 filling demonstrated a smoother characteristic. The roughness is uniformly distributed, suggesting consistent material removal across the surface. The processed surface displayed a finer, uniform texture with minimal roughness. The surface roughness profile showed an average roughness (Ra) of 0.16 μm for 6 × 30, indicating the moderate surface irregularity, while for 2 × 90 filling strategy Ra = 0.19 μm. For the further experiments, we only used 6 × 30 filling strategy, because it let to the highest depth and lowest surface roughness at the same time. As a practical aspect, also the complete duration of complex ablation process can be reduced by choosing this strategy.

### Effect of laser energy and pulse repetition rate on surface roughness

**Fig. 5:**
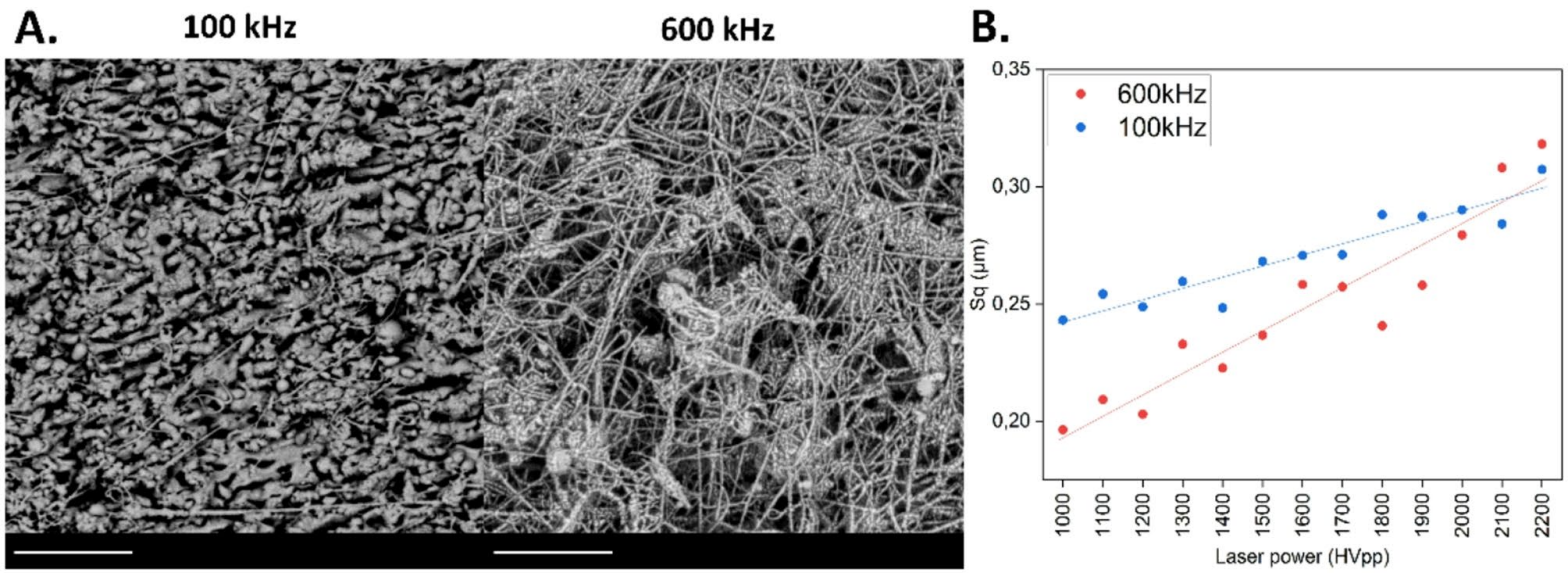
Effect of laser energy and pulse repetition rate on glass surface. (**A**) Exemplary SEM pictures of surface morphology obtained using filling strategy 2 × 90 at HVpp = 2200 V at different pulse repetition rates. The scale bar is 5 μm. (**B**) Surface roughness measured for filling strategy 2 × 90 at 100 kHz and 600 kHz. Coefficient of determination for studied cases equaled R2 = 0.89 for both 100 kHz and 600 kHz.

The repetition frequency of the laser pulses may influence the formation, morphology and characteristics of the structures on glass (Fig. 5A). 600 kHz repetition rate resulted in an increased density of glass particles and filaments due to cumulative thermal effects and higher material removal rates, while 100k Hz generated sharper, more spaced-out surface features due to less interaction between successive pulses. 100 kHz repetition rate, on the other hand, represented lower density of particles with cleaner structure features with minimal heat-affected zones, while at 600 kHz dense, closely spaced glass fibers were observed with some melted particles. A potential explanation is the accumulation of thermal energy at higher pulse repetition rates. Figure 5B shows comparison of surface roughness obtained at pulse frequencies of 100 kHz and 600 kHz. Using higher pulse frequency, the surface roughness could be reduced for the most laser power values. Due to continuous energy application, high overlap between pulses created a more continuous and uniform ablation, leading to significant roughness reduction (at HVpp = 1000 V from Sq = 0.243 μm to Sq = 0.197 μm). For 100 kHz the roughness tended to be higher compared to 600 kHz due to less continuous material removal and the formation of distinct ablation features. The topography showed more pronounced peaks and valleys due to less overlap between successive pulses, resulting in rougher texture. On the other hand, at 600 kHz repetition rate surface was more uniform, however the formation of micro-cracks in the subsurface region were noted with increasing laser power. Using higher pulse energies, the formation of cracks increased due to thermal stress and shock waves.

### Influence of the post chemical treatment on surface roughness

**Fig. 6:**
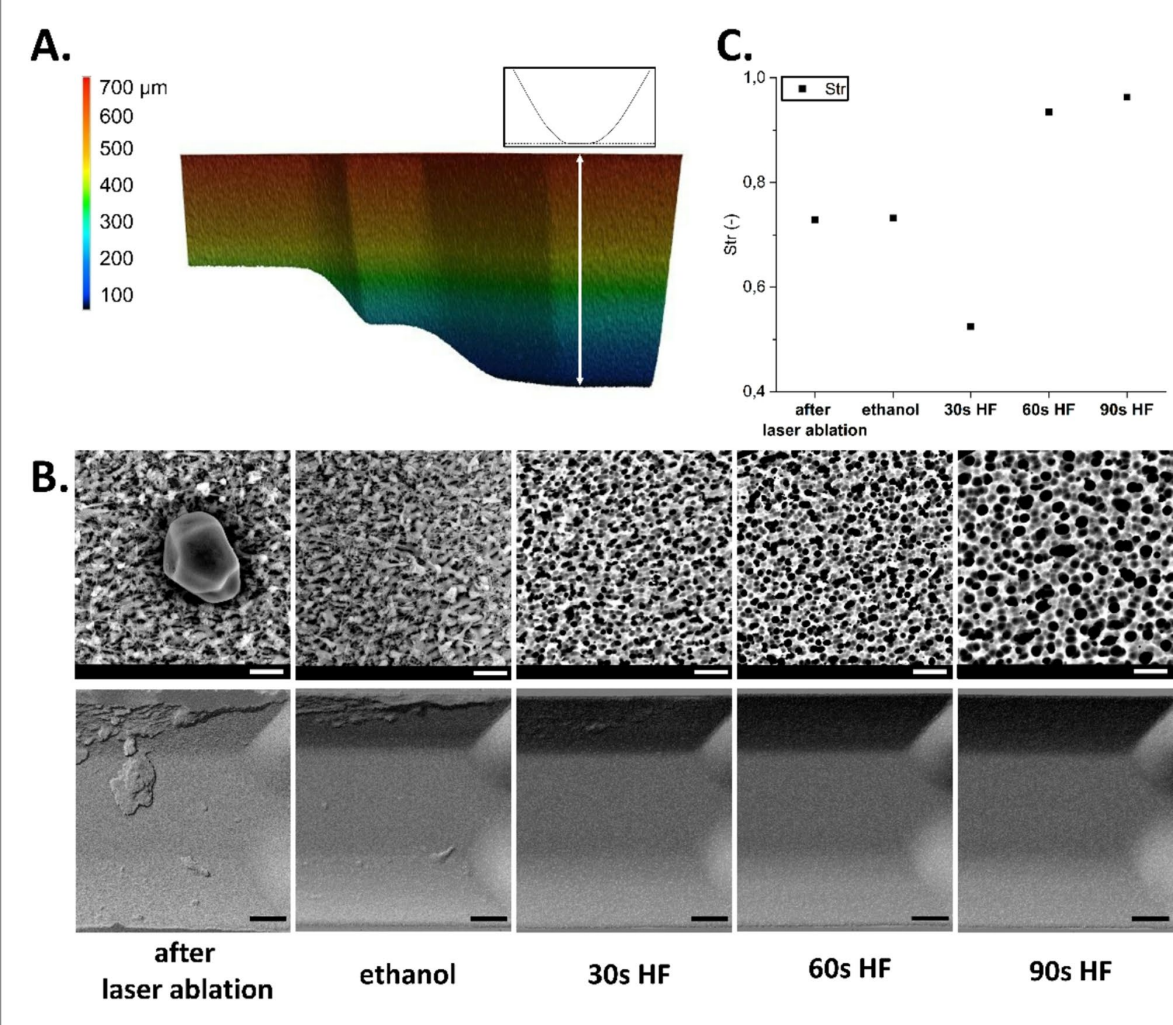
Post treatment after laser ablation including chemical cleaning. (**A**) Cross sectioned view of the evaluated complex system design with corresponding cross sectional line profile, that was used for roughness measurements. The roughness after 14 layers of 50 μm height ablation equals Sq = 1.73 μm. (**B**) SEM images (upper row) and DIC images (lower row) of laser structured parameter slots surfaces after subsequent chemical treatment steps. The first image shows the surface of the glass right after the laser ablation. For the second image, glass was cleaned in an ultrasonic bath filled with ethanol for 15 min to wash away the debris. For the following images, the glass was dipped in a glass-etching solution for 30 s, 60 s and 90 s resulting in typical surface pits. The scale bar is 5 μm for SEM and 200 μm for DIC images. (**C**) The roughness values (Str) on glass surfaces measured after the individual glass treatment steps. For a surface with a dominant lay, the Str parameter tends towards 0, whereas a spatially isotropic texture results in a Str close to 1. The progressing chemical treatments can selectively dissolve or wash away certain components of the glass, leading to specific surface features that can either increase or decrease surface roughness, depending on the treatment used.

With increasing number of ablation layers, femtosecond laser ablation generated debris and redeposited material around the ablated areas, what could increase surface roughness. In order to study the influence of chemical post-treatment on surface roughness in 3D structures, a channel with a depth of 700 μm (obtained with 14 layers ablation of 6 × 30 filling pattern) was evaluated (Fig. 6A). Directly after laser ablation, the machined surfaces were covered with glass filaments and particles (Fig. 6B). The subsequent chemical treatment improved the surface quality even after laser ablation. Combining ethanol solution with ultrasonic cleaning enhanced the removal of fine particles and residues, however any significant changes in the surface roughness have not been observed (Fig. 6C). After applying 30 s etching with the solution of hydrofluoric acid (HF), micro-scale debris left by the laser ablation process was etched away and a surface with a sharp and irregular round pits developed. Modifications in the surface morphology have been noticed, resulting in an increase of surface roughness. Str parameter appeared close to 0, indicating anisotropic surface texture with a clear dominant layer, in which the surface texture patterns are aligned. With the progressing etching time (60s and 90 s), etching solution selectively removed the roughened layer of glass, leading to a smoother surface. Str parameter was close to 1, indicating that the surface roughness declined and surface texture became isotropic.

Analyzing the discussed case, since the surfaces were sequentially processed in the cleaning process after laser ablation, the Str parameter most reliably represents the change in the examined surface. Additionally, due to the change in the geometric characteristics of the surface’s peaks and valleys rather than their height, the Str parameter allows for further assessment and evaluation. Due to the nature of the chemical treatment, the geometry of the original surface was modified by selective chemical treatment, and the surface became more isotropic—the Str parameter in the final phase of processing is closest to the value of 1.00 (Str = 0.96 after 90 s of HF etching). This is further confirmed by the attached SEM images (Fig. 6B) showing the opening of spaces between the individual peaks and valleys of the surface. Therefore, it can be significantly concluded that the directionality has changed substantially, transforming its character to isotropic.

**Fig. 7:**
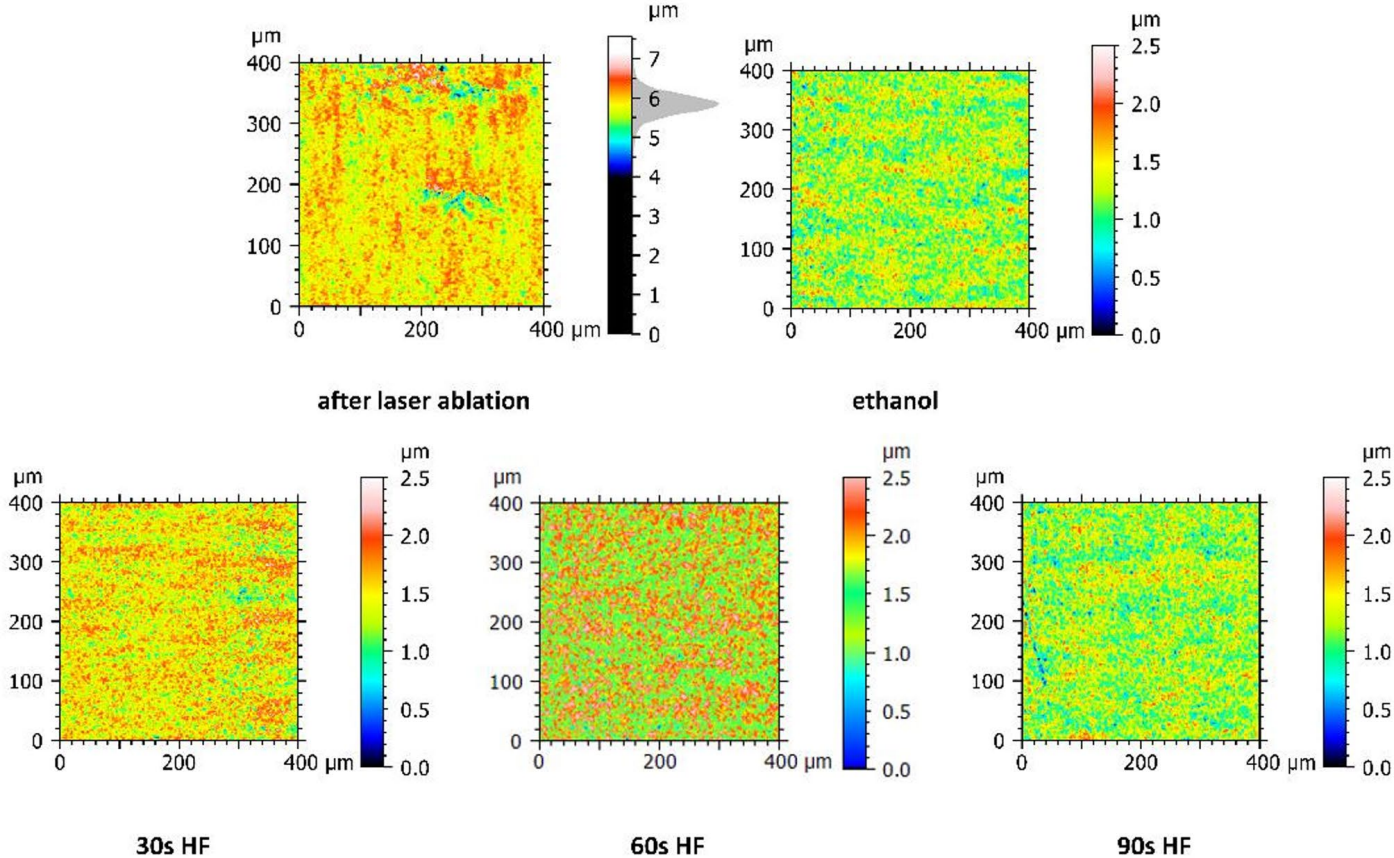
Surface topography after subsequent chemical treatment. With the colour scale for Z axis and the scale ranges, changes in surface topography indicated modifications in the surface topography after individual cleaning and etching steps. Note that roughness measured using AFM after single layer ablation of 50 μm (see Fig. 4A) were much lower than measured with CT scan after 14 layers that were needed to obtain the full depth 700 μm in the complex system.

Analysing surface topography (Fig. 7), a similar trend was observed. Directly after laser ablation, the surface features pronounced peaks and valleys, indicating roughness. Ethanol cleaning reduced exhibiting irregularities and unevenness, resulting in more uniform texture in all directions across its plane. After 30 s of HF etching surface topography displayed irregularities in height distribution, however continuing with subsequent HF cleaning, surface became smoother and indicated spatially isotropic texture. After 90 s of HF etching, the surface is smooth and even with minimal irregularities or roughness.

### Influence of the thermal post treatment on surface roughness

**Fig. 8:**
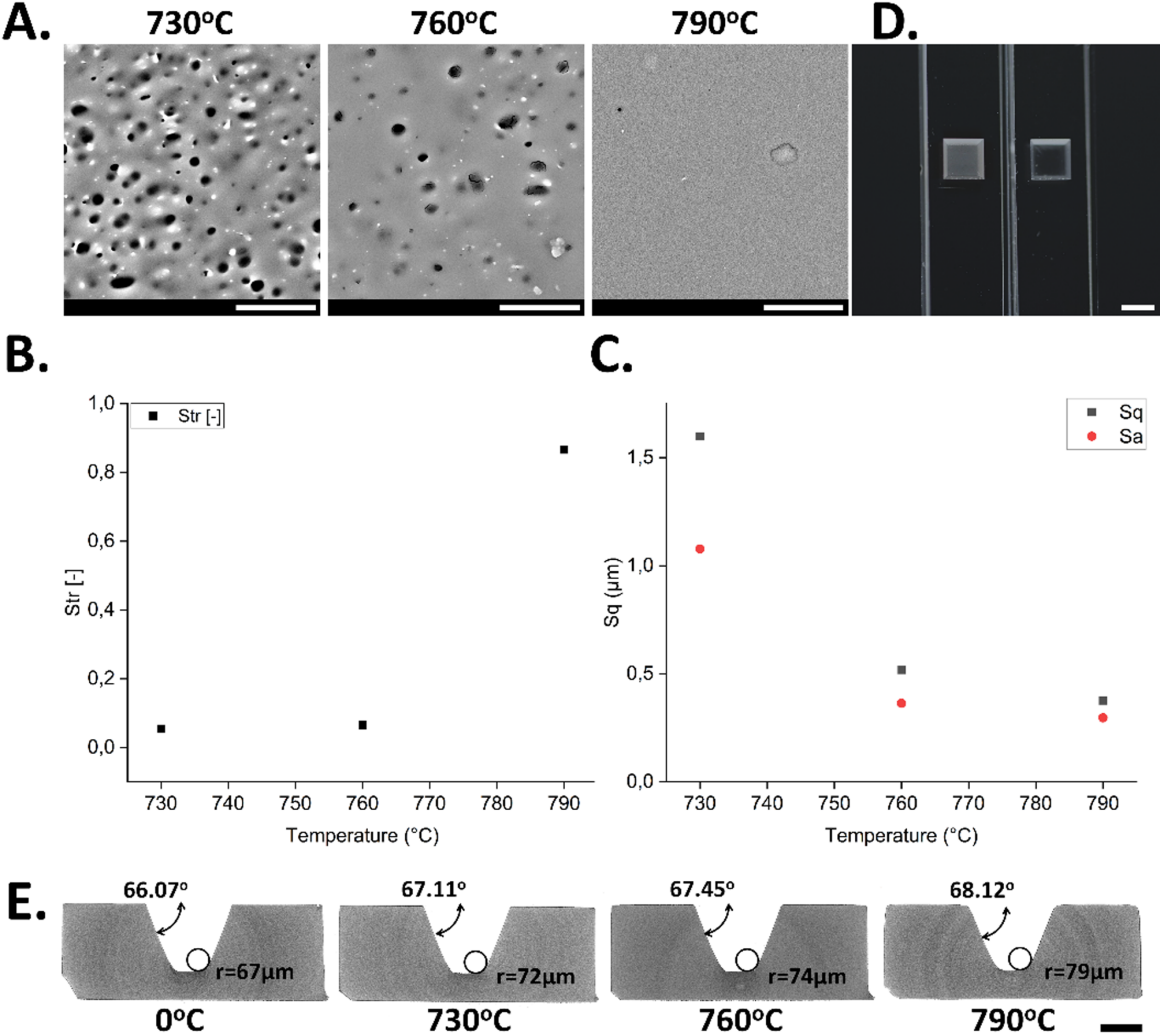
Effects of thermal annealing after femtosecond laser ablation. (**A**) Glass annealing process over different temperatures. The etched glass was tempered at 730 °C, 760 °C and 790 °C for 1 h resulting in an almost perfectly smooth surface. The scale bar is 10 μm. (**B**) Characteristic of surface roughness expressed by Sq (root mean square height) and Sa (average roughness) parameters after thermal treatment at different temperatures. (**C**) The roughness values (Str) on glass surfaces measured after annealing at different temperatures. (**D**) Surface structures before (left part of the image) and after combined chemical and thermal treatment (right part of the image). The scale bar is 2 mm. (**E**) Cross-sectional µCT scans (scale bar is 250 μm) of fabricated channels, showing the surface profile rounding obtained after annealing at 730 °C, 760 °C and 790 °C for 1 h together with corner radius and taper angle measurements.

Thermal post treatment can significantly influence the surface roughness of glass after femtosecond laser ablation. Heating the glass to a specific temperature below its melting point, maintaining that temperature for 1 h and then cooling it down gradually helped reduce surface porosity and roughness by gentle flow of the material and reduction of the surface energy through smoothing (Fig. 8A). The laser-induced material removal process inherently generates micro-cracks, molten fragments, and redeposited glass nanoparticles that are not fully removed by thermal annealing. Analyzing exemplary SEM pictures after thermal treatment at different temperatures, it could be recognized that direct annealing alone does not effectively eliminate surface debris or subsurface defects left from laser ablation. This is illustrated by few remaining glass particles (Fig. 8A at 760 °C and 790 °C). These residual particles are not only undesirable for microfluidic and optical applications—where surface smoothness and channel integrity are crucial—but also pose risks in biological applications due to potential cytotoxicity and contamination. Therefore, a combination of chemical and thermal post treatment was required. Annealing at temperatures of 730 °C, 760 °C and 790 °C for glass resulted in a notable reduction in surface roughness (Fig. 8B). The generated parameters underwent a comprehensive analysis of the impact of individual technological transitions on the obtained values of specific parameters. Standard parameters such as the arithmetic mean deviation of the surface roughness height from the reference plane Sa and the root mean square deviation of the surface roughness height from the reference plane Sq provided reliable quantitative roughness results, showing roughness reduction (from Sq = 1.60 μm and Sa = 1.08 μm at 730 °C to Sq = 0.38 μm and Sa = 0.30 μm at 790 °C). However, those parameters did not provide reliable quantitative roughness results and represent just a general measure of the texture forming the surface. They are insensitive to differentiating peaks, valleys, and spacing between various texture features. Therefore, Str parameter was analysed in order to study surface changes after annealing treatment at different temperature and measure the spatial isotropy or directionality of the surface texture (Fig. 8C).

At 730 °C and 760 °C (Str = 0.07) the surfaces with a dominant orientation of geometric features were observed and the Str parameter tended to approach a value of 0.00, while at 790 °C a spatially isotropic texture appeared and resulted in a Str of 0.87. Thus, the higher temperature of annealing used, the more the surface exhibits isotropic characteristics. With increasing annealing temperature, the etch pits started to disappear and the surface irregularities smoothed out due to glass flow (Fig. 8A). Str, Sa and Sq parameters have improved, which indicates not only a change in the surface structure geometry but also the height of valleys and peaks. Both temperature and duration of treatment must be optimized to avoid excessive flow or deformation of the glass. If the temperature is too high, it could cause unwanted melting or warping and 3D structures could start to deviate from their original shape. The reduction in surface roughness due to thermal treatment also improved the optical quality of the glass because unwanted surface scattering becomes suppressed (Fig. 8D). This is crucial for applications where optical quality surfaces are required, e.g. for high-resolution microscopy in microfluidic channels made of glass.

Figure 8E demonstrates the impact of annealing temperature on surface profile. Excessively high temperatures caused the laser-structured features to lose definition and become rounded. While the corner radius measured at the bottom increased from 67 μm to 79 μm with higher annealing temperature, the taper angle slightly increased from 66.07° to 68.12°. The latter is surprising at first, as a flattening was intuitively expected. The profile changes cannot be completely prevented, but the degree of rounding can be controlled by adjusting the annealing temperature.

### Optical performance

Transmittance (T) measures the fraction of light passing through a sample. With incident ($\:{P}_{i}$) and transmitted power ($\:{P}_{t}$) it is defined as

3 $$\:T=\left(\frac{{P}_{t}}{{P}_{i}}\right)\cdot\:100\%$$

**Fig. 9:**
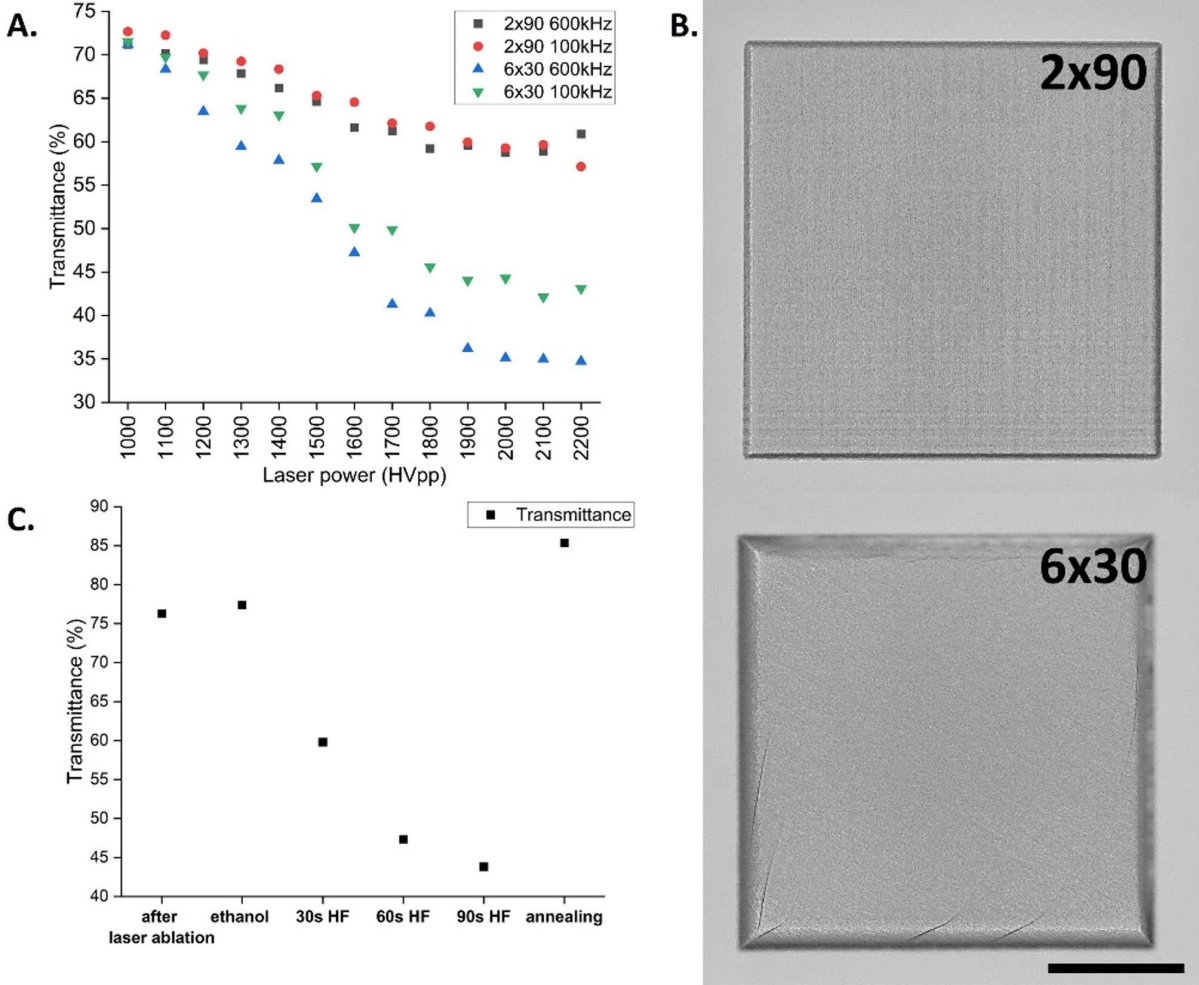
Optical transmittance of the borosilicate glass. (**A**) The influence of filling strategy and laser power on the glass transmittance measured for filling strategy with two and six scanning lines at 100 kHz and 600 kHz. (**B**) Optical appearance of ablated parameter slot using different density of scanning lines at 600 kHz. While 2 × 90 filling resulted in non-defect surface, 6 × 30 had visible surface and subsurface cracks. The scale bar is 500 μm. (**C**) Optical transmittance after post ablation chemical and thermal treatment. Annealing was carried out at 760^o^C for 1 h.

Figure 9A shows how the number of laser scanning lines influenced the light transmittance of glass. Each scanning pass leads to the interaction of the laser with the glass and changes the surface, affecting not only surface roughness but also transparency and clarity of the glass. While a plane glass wafer of 1.1 mm thickness revealed a transmittance of T = 92.9%, the ablated borosilicate glass gave values between 72.7% and 57.1% for the 2 × 90 filling strategy, while for the 6 × 30 results ranged from 71.5 to 34.7%. With more passes, the laser energy could accumulate, leading to greater thermal effects, stress and potentially more significant material alterations. As the number of passes and laser power increased, a significant reduction in transmittance was observed. Even though repeated passes could decrease surface roughness (Fig. 4B), due to the cumulative ablation effects they led to the formation of micro-cracks or other defects within the glass, scattering more light and thus decreasing transmittance (Fig. 9B). Figure 9C indicates that also subsequent post ablation chemical and thermal treatment have a crucial influence on the optical properties. The transmittance of plane glass wafer of 1.1 mm thickness (*T* = 92.9%) was reduced to *T* = 76.26% directly after laser ablation and slightly improved to *T* = 77.41% after routine ethanol cleaning with ultrasonic bath. Selective HF etching changed surface appearance (Fig. 6B), etching away the rough and damaged layers of glass but also introducing typical surface pits and modifying the surface morphology. These surface irregularities could scatter light, thereby degrading the optical clarity and a reducing the transmittance. Annealing at appropriate temperatures did effectively improve transmittance by allowing the glass to flow and partially heal these micro-cracks and defects, thereby enhancing the transmittance of the glass from *T* = 43.82% after 90 s HF to *T* = 85.35% which is quite close to *T* = 92.9% measured for non-structured glass. The improvement in optical transmittance is primarily attributed to reflow, defect healing and stress relaxation during the annealing. Thermal post-treatment facilitates the reduction of bulk defects within the material structure, which are known to scatter light and impede transmittance^33^. The processing conditions also contribute to the relaxation of internal stresses that accumulate during laser ablation. Such relaxation minimizes micro-crack propagation and reduces scattering interfaces.

## Conclusion

In femtosecond laser 3D microstructuring of glass, the laser parameters determine morphology and roughness of the created surfaces. In comparison to picosecond lasers (10^−12^ seconds) shorter pulse duration considerably can minimize the interaction time between the laser and the material, leading to less heat diffusion into the surrounding area and reduced surface roughness^34^. This study demonstrates that the final surface morphology and optical quality of glass substrates structured by femtosecond laser ablation are strongly influenced by laser parameters and post-processing treatments. Pulse energy is a fundamental parameter, affecting not only how much material is removed but also how much the adjacent material is damaged by a single pulse, which has decisive consequences for the surface roughness. Higher energy can increase surface roughness due to aggressive ablation and thermal effects. Lower energy provides finer control and smoother surfaces, but is less effective in material removal. Laser fluencies below 12.28 J/cm^2^ were found to minimize surface roughness while at the same time prevent damage in the bulk substrate material. The number of scan lines significantly influenced surface uniformity and combined with appropriate laser fluence plays crucial role in determining not only the surface roughness of the processed material, but also the amount of surface deformation^35^. Denser scan lines reduce surface roughness by progressively smoothing fluence inhomogenieties, while a lower density of scan lines might leave higher roughness. Chen et al.^36,37^ reported a subnanometer surface roughness with femtosecond laser polishing leading to a much lower ablation depth in the nanometer range. Fricke-Begemann et al.^38^ demonstrated ablation by F_2_-laser mask projection (157 nm wavelength) in fused silica glass, leading to roughness in the nanometer range at a depth of 1.5 μm. However, for most flexible structuring of 3D features, maskless fs-laser ablation is more practical. As ablation depth progresses, surface roughening accumulates. In our case the surface roughness increased from Sq ≤ 0.32 μm after 50 μm single layer ablation to Sq = 1.73 μm after multiple layer ablation, leading to 700 μm depth. Only with post treatments, Sq ≤ 0.5 μm could be reached for surfaces created in this depth. Optimized femtosecond laser parameters such as numbers of scanning lines or pulse repetition rate could significantly improve the transmittance of glass by reducing surface defects and thereby reducing the light absorption and scattering. The most significant enhancement in surface quality was achieved through the application of chemical and thermal post-treatments. These complementary processes effectively reduced residual roughness and surface defects left by laser structuring, further improving surface smoothness to near-optical quality. Optimized post-treatment conditions resulted in an impressive optical transmittance of up to 85.35% after multiple layer laser ablation (untreated glass transmittance *T* ~ 93%), indicating their critical role in achieving high-performance glass surfaces. The precise control of femtosecond laser parameters combined with targeted post-processing offers a robust strategy for tailoring surface morphology and optical properties. These findings provide practical guidelines for advancing glass-based optical and optofluidic devices, where superior surface quality and controlled roughness are essential for functionality and performance for which the recently published NeuroExaminer is only a prominent example.

## Electronic supplementary material

Below is the link to the electronic supplementary material.

Supplementary Material 1

## Data Availability

All data generated or analysed during this study are included in this published article (and its Supplementary Information files).
