# Supplementary material for: The route to microstructures with optical quality glass surfaces by fs laser ablation
Source: Sci Rep. 2025 Jul 14;15:25421. doi: 10.1038/s41598-025-11563-0 (PMC12260103; doi:10.1038/s41598-025-11563-0)
Supplement: Supplementary file 1 — Supplementary Material 1 [file 41598_2025_11563_MOESM1_ESM.docx]

Table 1: Parameters used for the femtosecond laser ablation of studied parameters slots within the base glass substrate.

|  |  | **100kHz** | | **600kHz** | |
| --- | --- | --- | --- | --- | --- |
| **High voltage of the pulse picker**  **(HVpp) [V]** | **Laser power [%]** | **Pulse energy [µJ]** | **Fluence [J/cm^2^]** | **Pulse energy [µJ]** | **Fluence [J/cm^2^]** |
| **1000** | 40 | 56.20 | 6.19 | 9.37 | 1.03 |
| **1100** | 45 | 66.04 | 7.27 | 11.01 | 1.21 |
| **1200** | 50 | 75.91 | 8.36 | 12.65 | 1.39 |
| **1300** | 55 | 85.71 | 9.44 | 14.29 | 1.57 |
| **1400** | 60 | 94.90 | 10.45 | 15.81 | 1.74 |
| **1500** | 65 | 103.47 | 11.40 | 17.25 | 1.90 |
| **1600** | 70 | 111.51 | 12.28 | 18.59 | 2.05 |
| **1700** | 75 | 118.42 | 13.04 | 19.74 | 2.17 |
| **1800** | 80 | 124.40 | 13.70 | 20.74 | 2.28 |
| **1900** | 85 | 129.00 | 14.21 | 21.5 | 2.37 |
| **2000** | 90 | 133.28 | 14.68 | 22.21 | 2.45 |
| **2100** | 95 | 136.43 | 15.03 | 22.74 | 2.50 |
| **2200** | 100 | 138.04 | 15.20 | 23.01 | 2.53 |
